# Clinical experience with a novel assay measuring cytomegalovirus (CMV)-specific CD4+ and CD8+ T-cell immunity by flow cytometry and intracellular cytokine staining to predict clinically significant CMV events

**DOI:** 10.1186/s12879-020-4787-4

**Published:** 2020-01-17

**Authors:** Ralph Rogers, Kapil Saharia, Aditya Chandrokar, Zoe F. Weiss, Kendra Vieira, Sophia Koo, Dimitrios Farmakiotis

**Affiliations:** 1 Division of Infectious Diseases, Department of Internal Medicine, Warren Alpert Medical School of Brown University, 593 Eddy Street, Gerry House 111, Providence, RI 02903 USA; 2 Division of Infectious Diseases and Institute of Human Virology, University of Maryland School of Medicine, Baltimore, MD USA; 3 Division of Infectious Diseases, Department of Internal Medicine, Brigham and Women’s Hospital, Harvard Medical School, Boston, MA USA

**Keywords:** CMV, Cytomegalovirus, Transplantation, Immunoassays, (val)ganciclovir

## Abstract

**Background:**

Cytomegalovirus (CMV) infection is one of the most common opportunistic infections following organ transplantation, despite administration of CMV prophylaxis. CMV-specific T-cell immunity (TCI) has been associated with reduced rates of CMV infection. We describe for the first time clinical experience using the CMV T-Cell Immunity Panel (CMV-TCIP), a commercially available assay which measures CMV-specific CD4+ and CD8+ T-cell responses, to predict clinically significant CMV events.

**Methods:**

Adult (> 18-year-old) patients with CMV-TCIP results and ≥ 1 subsequent assessment for CMV DNAemia were included at Brown University and the University of Maryland Medical Center-affiliated hospitals between 4/2017 and 5/2019. A clinically significant CMV event was defined as CMV DNAemia prompting initiation of treatment. We excluded indeterminate results, mostly due to background positivity, allogeneic hematopoetic cell transplant (HCT) recipients, or patients who were continued on antiviral therapy against CMV irrespective of the CMV-TCIP result, because ongoing antiviral therapy could prevent a CMV event.

**Results:**

We analyzed 44 samples from 37 patients: 31 were solid organ transplant recipients, 4 had hematologic malignancies, 2 had autoimmune disorders. The CMV-protection receiver operating characteristic (ROC) area under the curve (AUC) was significant for %CMV-specific CD4+ (AUC: 0.78, *P* < 0.001) and borderline for CD8+ (AUC: 0.66, *P* = 0.064) T-cells. At a cut-off value of 0.22% CMV-specific CD4+ T-cells, positive predictive value (PPV) for protection against CMV was 85% (95%CI 65–96%), and negative predictive value (NPV) was 67% (95%CI 41–87%).

**Conclusions:**

The CMV-TCIP, in particular %CMV-specific CD4+ T-cells, showed good diagnostic performance to predict CMV events. The CMV-TCIP may be a useful test in clinical practice, and merits further validation in larger prospective studies.

## Background

Cytomegalovirus (CMV) infection remains one of the most prevalent opportunistic infections (OI) following solid organ transplantation (SOT), and in individuals with hematologic malignancies or other immunocompromising conditions [1–3]. It is associated with significant morbidity due to its direct (CMV disease) and indirect (other OI, rejection, chronic allograft dysfunction) effects [1, 2, 4]. Prevention strategies after SOT consist of universal prophylaxis, preemptive therapy, or a combination of the two [1, 2]. However, these strategies have their respective pitfalls. For example, the optimal duration of antiviral prophylaxis is uncertain, varying from as short as 3 months to > 1 year [1, 2, 5]. Despite antiviral prophylaxis, patients may still develop CMV infection following discontinuation of prophylaxis [5–7]. In addition, there are risks of medication side-effects from antiviral prophylaxis, risk of drug resistance with prolonged antiviral prophylaxis, and the cost of antivirals can be prohibitive. Preemptive monitoring strategies can be inconvenient due to the need for serial viral load (VL) monitoring (every 1–2 weeks) [1, 2, 6–8].

The development of CMV infection and severity of CMV disease are largely influenced by the ability of the immune system to control viral replication. This generally requires intact humoral and cell-mediated immune responses, of which the latter is a frequent target of immunosuppressant therapy in SOT recipients. Despite treatment with T-cell inhibiting medications, most SOT recipients do not develop CMV infection, which suggests many individuals are able to maintain T-cell responsiveness against CMV. Much effort has been expended searching for a good measure of immune competency against CMV, including both global (non-pathogen specific) and CMV-specific assays [2]. An ideal diagnostic test that could provide a measure of immune competency to control CMV infection might allow for personalized anti-CMV care.

To this end, several CMV-specific assays evaluating cell-mediated immunity (CMI) have been developed: The Quantiferon®-CMV assay exposes whole blood to 21 CMV epitopes. Interferon gamma (IFN-γ) released from activated CD8+ T-cells is then quantified via ELISA, allowing for a measure of CMV-specific CD8+ (but not CD4+) T-cell response [8–16]. The T-Track CMV® and T-SPOT®-CMV assays first isolate peripheral blood mononuclear cells (PBMC), then expose them to a variety of CMV antigens or lysates; the resulting IFN-γ is quantified via ELISpot allowing for a measure of aggregate CMV-specific CD4+/CD8+ T-cell and NK-cell response, but not its individual components [17–26]. None of these assays are commercially available in the United States (US).

The Viracor® CMV T-cell Immunity Panel (CMV-TCIP) is the first commercially available assay measuring CMV-specific CMI in the US. CMV-TCIP is a flow cytometric assay that measures %CMV-specific CD4+ and CD8+ T-cells separately, following stimulation of whole blood with CMV peptides and lysates [27]. In this study, we describe for the first time patient-level experience with the CMV-TCIP, and assess the potential utility of CMV-specific CD4+ and CD8+ T-cell responses in predicting clinically significant CMV events.

## Methods

### Patients

We retrospectively studied adult (> 18-year-old) patients with CMV-TCIP results and ≥ 1 subsequent CMV-VL at Brown University and the University of Maryland Medical Center (UMMC)-affiliated hospitals, between 4/2017 and 5/2019. Clinically significant CMV events were defined as CMV DNAemia (> 1000 copies/mL [28]) or any detectable VL with symptoms suspicious for CMV infection by clinician assessment, prompting initiation of treatment.

We excluded from analysis indeterminate (due to background positivity) results, allogeneic hematopoetic cell transplant (HCT) recipients, or patients who were continued on antiviral therapy against CMV, irrespective of the CMV-TCIP result, since ongoing antiviral therapy could prevent a CMV event.

As a rule, CMV IgG donor seropositive/recipient negative (D+/R-) kidney and heart transplant recipients receive 6 months, CMV R+ kidney and heart transplant and all liver transplant recipients 3 months of valganciclovir prophylaxis. The duration of prophylaxis is extended in lung transplant recipients (D+/R-: lifelong, R+: 6–12 months). Patients with CMV infection have CMV-VL tested every 1–2 weeks for the first 2 months after discontinuation of valganciclovir. All patients with CMV-TCIP results included in analyses had ≥1 subsequent CMV-VL and were followed clinically for 6 months after discontinuation of valganciclovir, or after the CMV-TCIP for patients who did not receive antiviral medications. CMV-TCIP were ordered at the time (within a week) of discontinuation of valganciclovir or low-level CMV viremia for patients not receiving valganciclovir. The study was approved by both Institutional Review Boards, in agreement with the Declaration of Helsinki (Brown IRB study approval # 1346550, UMMC IRB study approval # HP-00082131).

### CMV-TCIP

Patient specimens were sent to the Viracor® laboratory for CMV-TCIP per protocol: Whole blood samples were stimulated with either CMV pp65 peptide mix (JPT, 138 peptide mix) or CMV grade 2 antigen mixture (Microbix Biosystems). Additional aliquots of each sample were incubated without stimulation (negative control) or with a nonspecific mitogen, *Staphylococcus aureus* Enterotoxin Type B (SEB) (positive control). Samples were stimulated overnight in a 37 °C incubator with 5% CO2 and in the presence of Brefeldin A (BD Biosciences) and co-stimulatory CD28/49d antibody (BD Biosciences). Following overnight incubation, samples were fixed and permeabilized for intracellular cytokine staining (ICS). Fluorescent-tagged antibodies (BD Biosciences) used to stain cell surface markers included: anti-CD3+ (CD3-PerCPCy5.5), CD4+ (CD4-Pacific Blue), and CD8+ (CD8-APC-Cy7). Fluorescent-tagged antibodies (BD Biosciences) used to measure T-cell activation in response to antigen stimulation included: CD69 (CD69-PECy7) and interferon-γ (IFN-γ FITC). Following ICS, samples were analyzed by flow cytometry (FC) using a Beckman Coulter Navios Flow Cytometer.

**Fig. 1:**
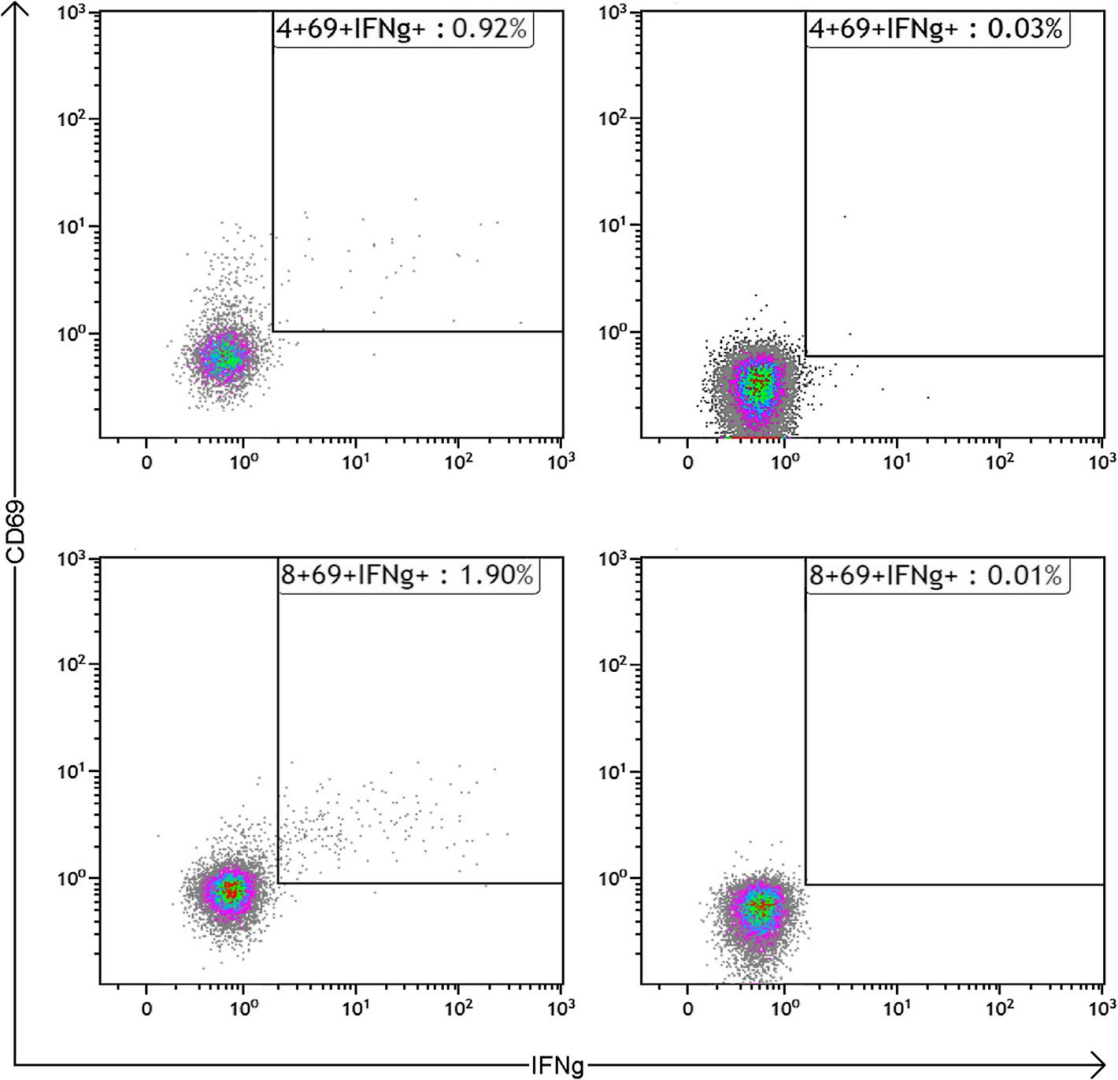
CMV-TCIP flow images for CD4+ (top) and CD8+ (bottom) populations demonstrating CMV-specific response (left) or no response (right). IFNG: Interferon-gamma

Based on data used to develop the assay from CMV IgG+ immunocompetent individuals, CMV-specific CD4+ or CD8+ responses > 0.2% are considered indicative of CMV-specific CMI (Fig. 1). Specifically, when analyzing a population of healthy adults, 100% of CMV seronegative adults had CMV-specific CD4+ or CD8+ responses < 0.2%, whereas 91% of CMV seropositive adults had CMV-specific CD4+ or CD8+ responses > 0.2% [27].

### Statistical analyses

Data are presented as mean (standard deviation-S.D.) or median (25th–75th inter-quartile percentile range (IQR)) for variables of normal or non-normal distribution (by Kolmogorov-Smirnoff test), respectively. We compared continuous variables with Student’s t, or Mann-Whitney tests for independent samples, and paired t or Wilcoxon signed-rank tests for related samples. We compared categorical variables between groups using χ^2^ or Fisher’s exact tests. We generated receiver-operating characteristic (ROC) curves to summarize diagnostic performance of tests in predicting protection from CMV. We calculated sensitivity, specificity, positive (PPV, protected against CMV) and negative (NPV, CMV event) predictive values, and their 95% confidence intervals (95%CI), for cut-off values that were closest to assay validation cut-off value (0.2%). Correlations were assessed by Pearson coefficient after logarithmic transformation of variables with a non-normal distribution. We used a two-tailed α threshold of ≤0.05. Analyses were performed with SPSS statistical software, version 24, IBM Corporation (Armonk, NY, USA) and Medcalc statistical software version 12.5.0.0. (Medcalc Software, Ostend, Belgium).

## Results

### Patients

We identified 59 CMV-TCIP samples (Brown: 47, UMMC: 12). We excluded 8 (Brown 5, UMMC 3) indeterminate assay results (7 with high background response, one (UMMC) with CD69+ but no interferon upregulation); we also excluded 5 results from Brown, where the clinicians continued valganciclovir, one pediatric patient (also with high CD8+ background response), one allogeneic HCT recipient.

We analyzed 44 samples (Brown 35, UMMC 9) from 37 patients (Brown: 28, UMMC: 9). Thirty-one (31) were SOT recipients, of which the majority (20/31) were kidney transplant recipients. Four patients had hematologic malignancies (2 multiple myeloma, 1 cutaneous T-cell lymphoma treated with alemtuzumab, 1 diffuse large B-cell lymphoma and HIV) with CMV DNAemia. One patient had autoimmune colitis treated with high-dose steroids and infliximab, after which he developed proven CMV colitis. Another patient had systemic lupus erythematosus and CMV pneumonia. Clinical features of study patients at the time of CMV-TCIP are summarized in Table 1.

**Table 1:** Characteristics of the study patient population at the time of CMV-TCIP

Parameter	Patients (*N* = 37)	CMV events (*N* = 16)	No CMV events (*N* = 28)
Age (years) (mean ± SD)	56.4 ± 15.4	51.5 ± 17.7	57.9 ± 13.6
Women (%)	15 (40.5)	9 (56.3)	12 (42.8)
SOT (%)	31 (83.7)	15 (93.7)	23 (82.2)
Kidney (%)	20 (54.1)	9 (56.3)	15 (53.6)
Kidney-pancreas (%)	2 (5.4)	1 (6.3)	1 (3.5)
Heart (%)	6 (16.2)	4 (25)	5 (17.9)
Lung (%)	2 (5.4)	1 (6.3)	1 (3.5)
Liver (%)	1 (2.7)	0 (0)	1 (3.5)
High-risk for CMV (CMV D+/R-) (%)	20 (54.1)	12 (75)	14 (50)*
Time from transplant (months) (median, IQR)	11.9 (7.1–17.2)	10.2 (8.3–18.5)	12 (7–16.7)
Induction immunosuppression			
Thymoglobulin (%)	6 (16.2)	4 (25)	5 (17.9)
Alemtuzumab (%)	6 (16.2)	2 (12.5)	4 (14.2)
Basiliximab (%)	16 (43.2)	8 (50)	11 (39.3)
None (%)	1 (2.7)	0 (0)	1 (3.5)
Unknown (%)	2 (5.4)	1 (6.3)	2 (7.1)
Maintenance immunosuppression			
2 agents (%)	12 (32.4)	6 (37.5)	9 (32.1)
3 agents (%)	19 (51.3)	9 (56.3)	14 (50)
High-dose mycophenolate (%)^a^	5 (13.5)	2 (12.5)	4 (14.2)
Tacrolimus trough level (ng/dL) (mean ± SD)	8.2 ± 4.2	8.6 ± 4.9	7.9 ± 3.8
Prednisone > 5 mg daily (%)	15 (40.5)	4 (25)	13 (46.4)
Non-SOT (%)	6 (16.2)	1 (6.3)	5 (17.8)
ALC (10^9^/Lt) (mean ± SD)	1 ± 0.79	1 ± 0.88	1 ± 0.76

Data are presented as absolute number (% within column) for categorical variables and mean ± standard deviation (SD) or median (interquartile range, IQR) for continuous variables with a normal or non-normal distribution, respectively

*ALC* Absolute lymphocyte count, *CMV D+/R-* Donor positive-recipient negative CMV seropositivity status, *SOT* Solid organ transplant

*P = 0.1

^a^Mycophenolic acid 720 mg or mycophenolate mofetil 1 g twice daily

### CMV events

We captured 16 CMV events (36%) in 14 patients (38%). The following parameters (at the time of CMV-TCIP) were not significantly different in patients who had CMV events compared to those who did not: Age, sex, organ transplanted, time from transplant, induction immunosuppression, 3-drug maintenance immunosuppression, high-dose mycophenolate (mycophenolic acid 720 mg or mycophenolate mofetil 1 g twice daily), prednisone dose (> 5 mg daily), tacrolimus trough level, absolute lymphocyte count (ALC). As expected, more D+/R- SOT recipients had CMV events (75%), compared to other patients (25%, *P* = 0.1).

**Fig. 2:**
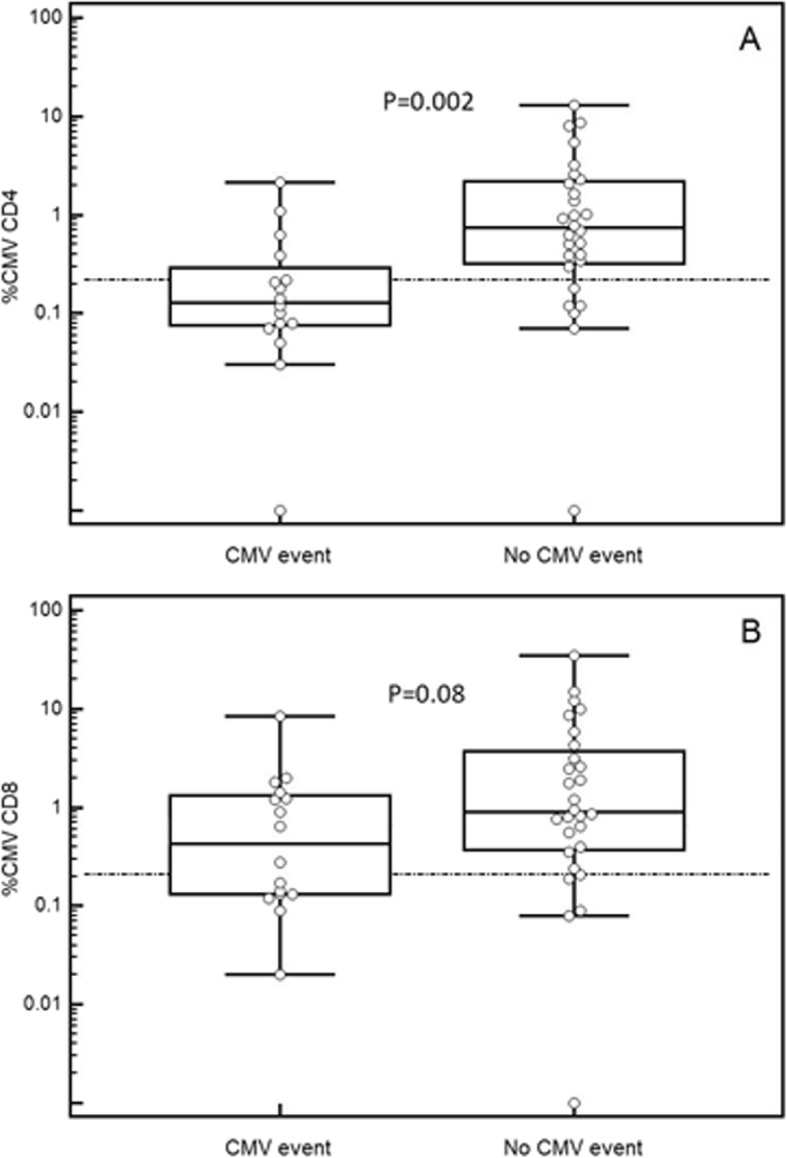
%CMV-specific CD4+ (**a** Mann-Whitney *P* = 0.002) and CD8+ (**b** Mann-Whitney *P* = 0.08) T-cells in patients who subsequently had CMV events compared to those who did not (median, IQR, 1.5xIQR). Horizontal reference lines correspond to cut-off values of 0.22% (CMV-specific CD4+ T-cells), and 0.21% (CMV-specific CD8+ T-cells), as explained in the text

The %CMV-specific CD4+ cells was significantly lower in patients with CMV events (median 0.13, IQR 0.08–0.3) compared to those without CMV events (0.73, 0.32–2.19, *P* = 0.002). The %CMV-specific CD8+ cells was also lower in patients with CMV events (0.46, 0.13–1.33) than those without CMV events (0.9, 0.37–3.75), though these results did not reach statistical significance (*P* = 0.08) (Fig. 2).

The %CD4+ response to SEB (positive control) was lower in patients with CMV events than those without CMV events (1.63, 0.66–3.71 vs 2.83, 1.97–4.19, *P* = 0.08), though results did not reach statistical significance. A similar trend was also noted for %CD8+ response to SEB (3.44, 1.54–9.47, vs 6.47, 3.28–10.27, *P* = 0.2).

### Diagnostic performance of CMV-TCIP

**Fig. 3:**
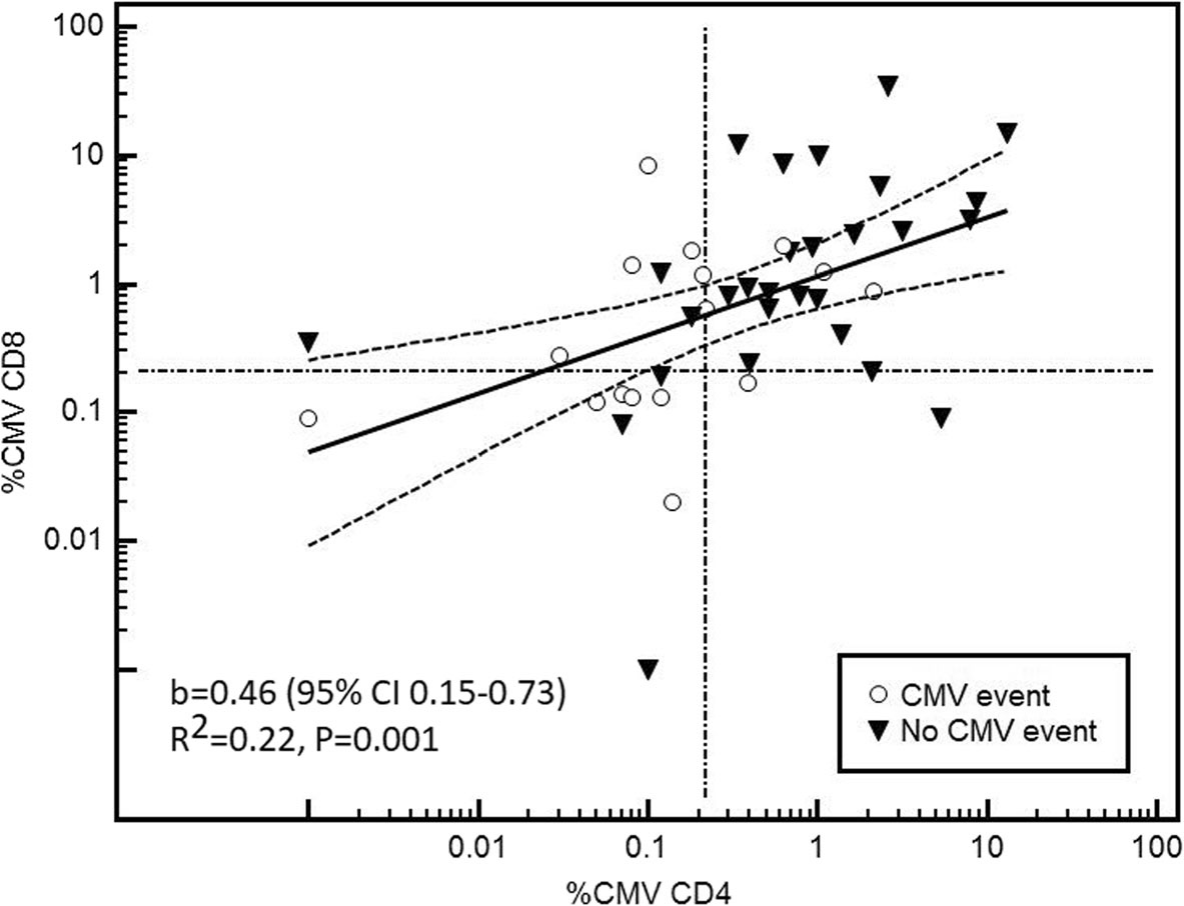
Scatterplot diagram and linear regression line (95%CI) for the correlation between %CMV-specific CD4+ and CD8+ T-cells (b: slope coefficient). The reference lines correspond to cut-off values of 0.22 and 0.21% CMV-specific CD4+ and CD8+ T-cells, respectively, as explained in the text

We found a strong positive correlation between %CMV-specific CD4+ and CD8+ T-cells (*r* = 0.47 (95% CI 0.2–0.65), *P* = 0.001, Fig. 3).

**Fig. 4:**
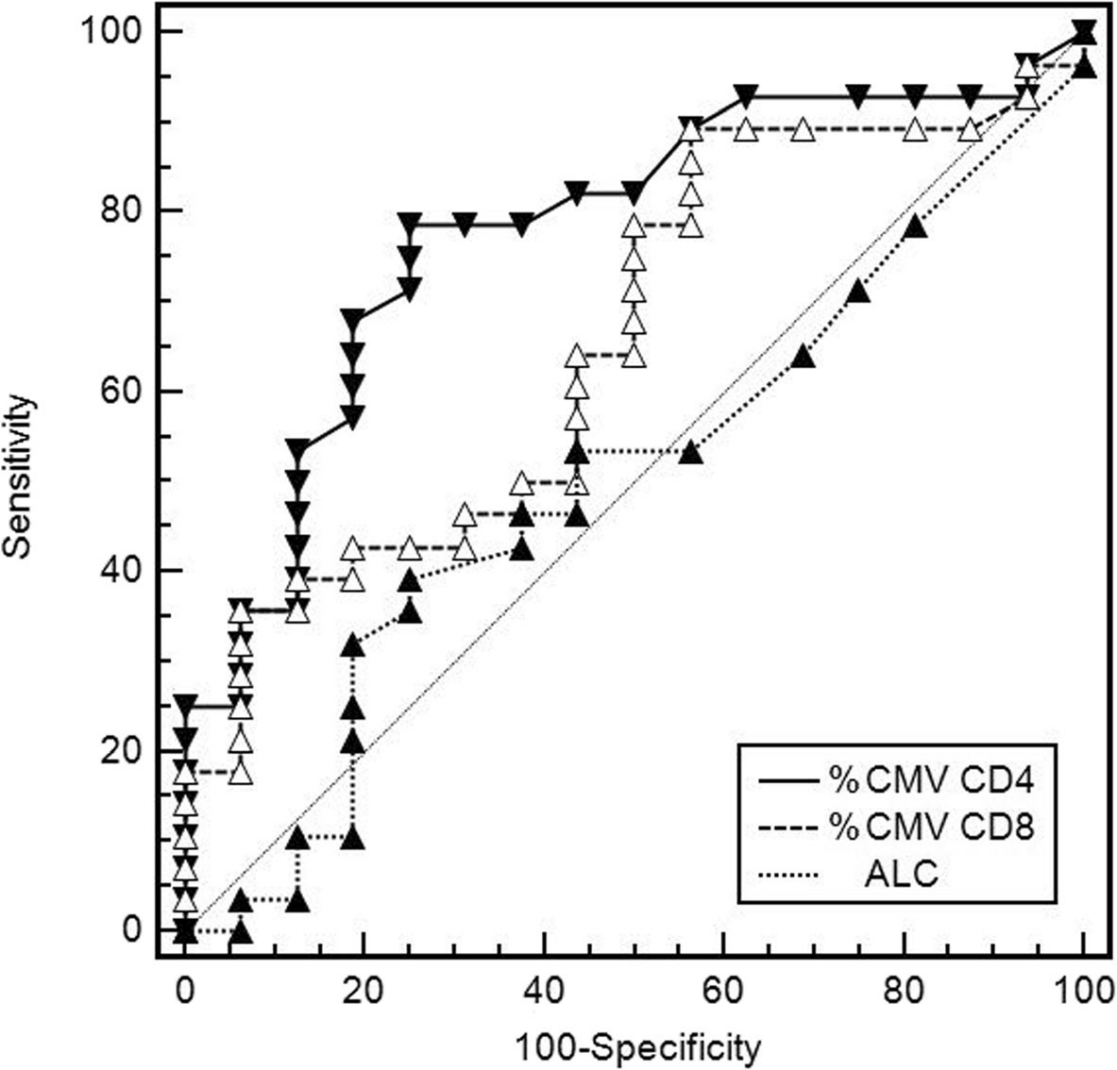
Receiver-operating characteristic (ROC) curves of %CMV-specific CD4+, CD8+ T-cells and the absolute lymphocyte count (ALC) as tests to predict subsequent CMV events. The diagonal line corresponds to area under the curve (AUC) of 0.5

The CMV-protection ROC Area Under the Curve (AUC) was significant for %CMV-specific CD4+ (AUC 0.78, 95%CI 0.63–0.89, *P* < 0.001) and nearly reached statistical significance for CD8+ T-cells (0.66, 0.5–0.79, *P* = 0.064), but not for any other parameters, including absolute ALC (0.5, 0.35–0.66, *P* = 0.96; *P* = 0.008 compared to the ROC AUC for %CMV-specific CD4+) (Fig. 4).

We calculated sensitivity, specificity, PPV and NPV for the %CMV-specific cut-off value from ROC curve analysis that was closest to the cut-off of 0.2%, which was deemed to be indicative of CMV-specific CMI during assay development, based on the response of CMV IgG+ healthy individuals [27]. Specifically, at a cut-off value of > 0.21% CMV-specific CD8+ T-cells, sensitivity for protection against CMV was 82% (95%CI 63–94%), specificity 44% (20–70%), PPV 72% (53–86%), NPV 58% (28–85%). At a cut-off value of > 0.22% CMV-specific CD4+ T-cells, sensitivity was 79% (59–92%), specificity 75% (48–93%), PPV 85% (65–96%), NPV 67% (41–87%) (Fig. 2).

**Fig. 5:**
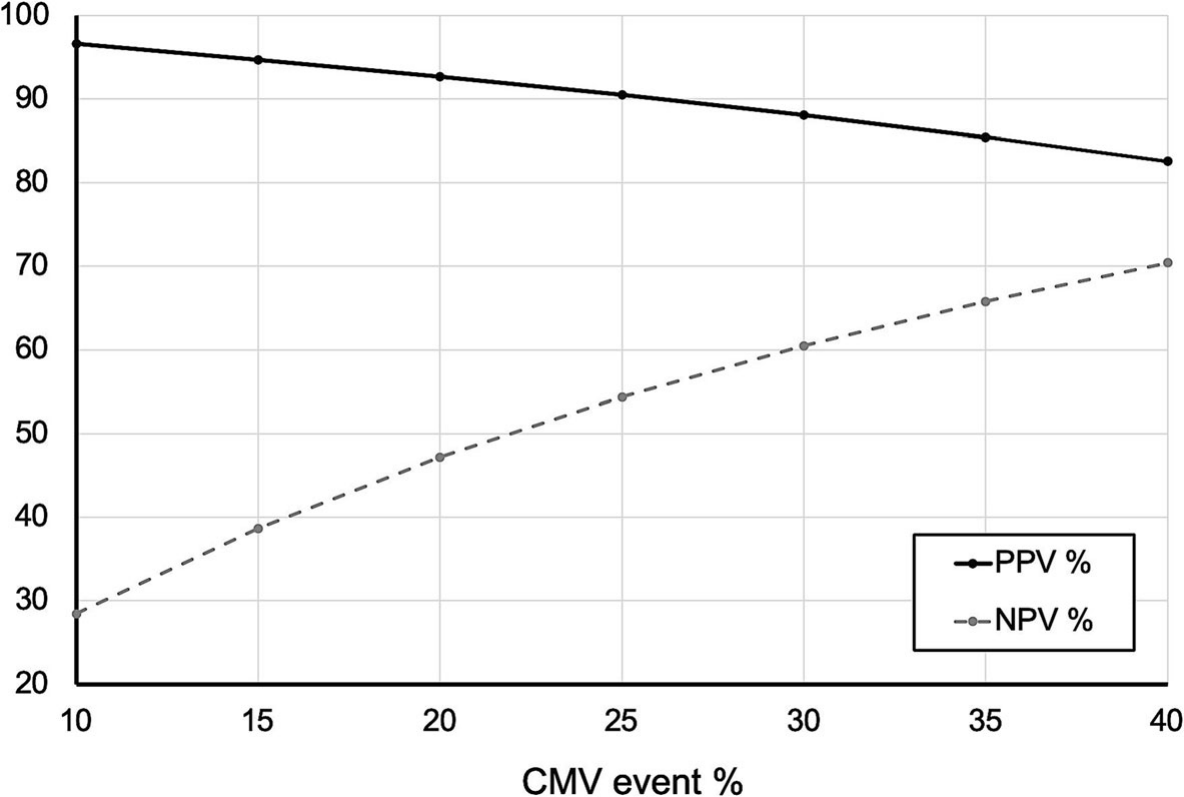
Positive (PPV) and negative (NPV) predictive values of %CMV-specific CD4+ T-cells for different frequencies (pre-test probabilities) of CMV events

As the % of CMV events in our study was rather high, we plotted positive (PPV) and negative (NPV) predictive values of %CMV-specific CD4+ T-cells for different frequencies of CMV events. For a pre-test probability between 15 and 25% (the CMV event rate in most studies [6, 8, 11, 29]), we estimated a PPV between 90 and 95% and NPV between 39 and 54% (Fig. 5).

The peak CMV-VL after CMV-TCIP was significantly higher when CMV-specific CD4+ T-cell response was ≤0.22% vs. > 0.22% (median 700, IQR 219–1800 vs. 0, 0–600 copies/mL, *P* = 0.005) but not when CMV-specific CD8+ T-cell response was ≤0.21% vs. > 0.21%, where the difference did not reach statistical significance at the *P* < 0.05 level (median 1037, IQR 0–4800 vs. 231, 0–644 copies/mL, *P* = 0.13).

### Repeat CMV-TCIP

**Fig. 6:**
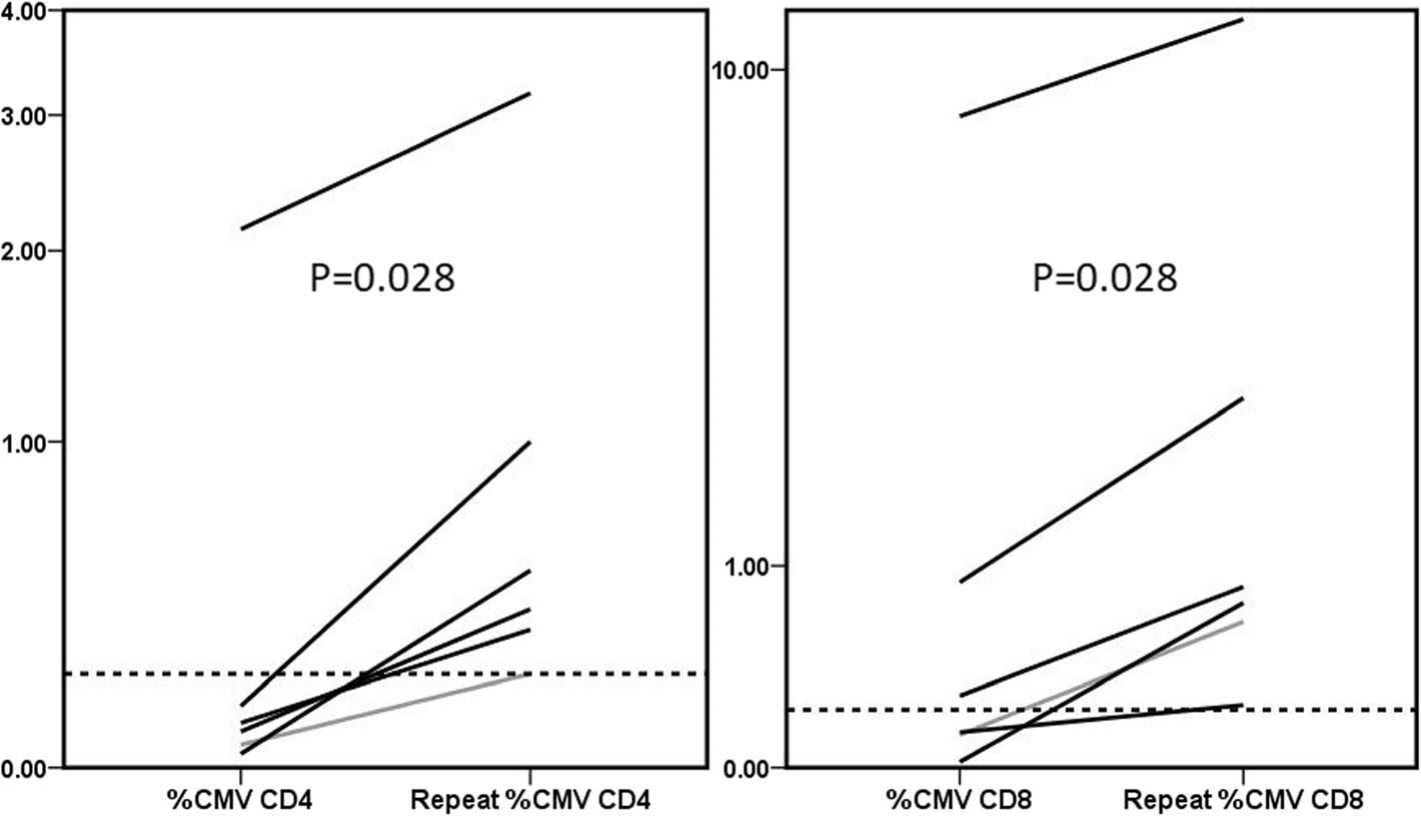
Significant increases in the % of CMV-specific CD4+ (left) and CD8+ (right) T-cells with time, after the initial CMV event (Wilcoxon rank sum *P* = 0.028). Five of six patients (black lines), who all had > 0.22% CMV-specific CD4+ T-cells on repeat testing, did not experience subsequent CMV events after discontinuation of valganciclovir (Reference lines: 0.22 and 0.21% for CD4+ and CD8+, respectively, as explained in the text)

Six patients had repeat CMV TCIP following their initial CMV event. All six were SOT recipients and 5 were high-risk for CMV (CMV D+/R-). We found a significant increase in %CMV-specific CD4+, %CMV-specific CD8+ (Fig. 6) and %SEB CD4+ responses (*P* = 0.028); the increase in %SEB CD8+ response was not statistically significant (*P* = 0.345). Five patients, all with > 0.22% CMV-specific CD4+ T-cells on repeat testing, did not experience subsequent CMV events after discontinuation of valganciclovir.

### False positive results

Four (4) CMV R+ transplant recipients with positive %CMV-specific CD4+ responses (3 of whom also had positive CMV-specific CD8+ responses, Figs. 2 and 3) had subsequent CMV events, defined as DNAemia and initiation of treatment with valganciclovir by clinicians. Two patients (both renal transplant recipients) were restarted on valganciclovir for asymptomatic low-level CMV DNAaemia (CMV-VL were 600 and 534 copies/mL).

One heart transplant recipient with positive CMV-specific CD4+ and CD8+ responses was restarted empirically on valganciclovir for CMV DNAemia (1100 copies/mL) and oral ulcers after stopping primary prophylaxis for CMV. A second CMV-VL was also detectable at 700 copies/mL prior to starting treatment. No testing was performed to evaluate etiology of oral ulcer as it was presumed to be related to CMV. Valganciclovir was eventually stopped following resolution of oral ulcers and after achieving undetectable CMV-VL. There was no recurrence of CMV DNAemia. Interestingly, the oral ulcers recurred, and after appropriate testing (PCR), were found to be due to herpes simplex virus.

The fourth patient had a positive CMV-specific CD4+ but negative CD8+ response and had chronic diarrhea for 2 years after kidney transplant. Colonoscopy showed rare cells that were positive for CMV by immunochemistry, without cytopathic changes. He had low-level (< 1000) CMV DNAemia and was treated with valganciclovir achieving undetectable CMV-VL, but the frequency of his diarrhea did not change.

## Discussion

In this study, we found a strong correlation between the results of the CMV-TCIP, specifically low CMV-specific CD4+ T-cells measured by ICS and FC, and subsequent CMV events. The association between CMV events and CMV-specific CD8+ T-cells did not reach statistical significance, although *P*-value was 0.06 (Figs. 2 and 4). In patients with repeat CMV-TCIP, CMV-specific CMI became stronger over time, facilitating discontinuation of valganciclovir (Fig. 6). Our report provides the first real-world data on the predictive value of this commercially available assay, that is supportive of its potential clinical utility.

A diagnostic test of immune competency against CMV can be utilized in different scenarios: at the end of primary prophylaxis, to determine if extended prophylaxis or close VL monitoring might be of benefit [10–16, 18, 22, 25]; at the end of treatment of CMV infection, to support the need for secondary prophylaxis [6, 8]; finally, in patients with asymptomatic CMV DNAemia, to determine if antiviral treatment is truly indicated [12, 13, 17, 23, 29]. Herein, the CMV-TCIP performed well in a relatively small (but comparable in size to other similar studies [8, 12, 29, 30]) case series, including all three potential scenaria. Larger-scale prospective studies should evaluate clinical utility of the assay in each.

Over the last decade, there has been growing interest in the development and bedside implementation of CMV-CMI assays. The Quantiferon®-CMV assay can predict late-onset CMV disease after primary prophylaxis [10–16] and spontaneous clearance of CMV DNAemia [12, 13, 29]; it has also been used in two interventional studies to guide primary [16] or secondary (after treatment for a CMV event) [8] prophylaxis. ELISPOT-based CMV-CMI assays (T-Track CMV® and T-SPOT®-CMV) can also help predict CMV events [17–26]. It should be noted though that, at the time of this manuscript, these assays are limited to research use only in the US.

When comparing CMV-CMI assays, one study demonstrated that an ELISPOT-based assay performed similar to the Quantiferon®-CMV assay [31], while a recent meta-analysis suggests that ELISPOT-based CMV-CMI testing might perform better than Quantiferon®-CMV in predicting CMV events [32]. While the CMV-TCIP has been studied less than the above assays, the methods on which it is based (ICS/FC) have served as the gold-standard of immunoassays for years [30, 33]. Also, the predictive performance of the test in our study was comparable to previous reports, especially after adjustment for pre-test probability (Fig. 5).

The relevance of individual CD4+/CD8 + T-cell subpopulations in the immune response to CMV infection in SOT recipients has been extensively studied, primarily via ICS/FC [30]. One early study of renal transplant recipients showed that presence of CMV-specific CD4+, not CD8+, T-cells was protective against development of CMV disease [34]. On the contrary, a study of heart and lung transplant recipients suggested that CMV-specific CD8+, not CD4+, T-cells were protective [35]. More recently, a study of CMV-specific T-cell subpopulations in renal transplant recipients demonstrated that low pre-transplant CD8+ T-cells, low post-transplant day (PTD) 15 CD4+ or CD8+ T-cells, and low PTD60 and PTD180 CD4+ T-cells were predictive of subsequent CMV events [36]. Given the complex interactions between subpopulations of the cellular immune system, it is likely that both CMV-specific CD4+ and CD8+ T-cells play a role in the immune response to CMV infection [30].

A limitation of the Quantiferon®-CMV assay is that is seems to be more skewed towards CD8+ response. ELISPOT-based CMV-CMI testing, while measuring both CD4+ and CD8+ responses, does not provide detailed analysis of the individual components. The CMV-TCIP is the only clinical test of CMV-specific CMI to date that analyzes CD8+ and CD4+ T-cell responses separately. CMV-specific CD4+ T-cells are necessary to generate a pool of memory cytotoxic CD8+ T-cells, that can potentially prevent disease, by controlling recurrent CMV viremia, in the absence of antiviral medication [30, 37]. This mechanism could account for the strong association of %CMV-specific CD4+, more than CD8+ T-cells with protection against CMV in our study, and the better performance of ELISPOT-based CMV-CMI testing, compared to the Quantiferon®-CMV in predicting CMV events [32]. Importantly, there is also evidence that CD4+ T cells have direct antiviral properties against CMV and play an essential role in abrogating reactivation and controlling primary CMV infection [38–40]. Given the importance of both CD4+ [4, 30, 34, 36, 41, 42] and CD8+ [35, 41, 42] T-cells in the immune response to CMV, and the variety of clinical scenarios in which CMI assays may be used, detailed information regarding both CD4+ and CD8+ specific responses could be of clinical utility.

Recently, investigators have also examined measures of global immunity to predict subsequent CMV events, focusing on inexpensive tests, that most clinicians order routinely [6, 43]. One such study in SOT recipients showed that the absolute lymphocyte count (ALC) at the completion of CMV treatment was independently associated with risk for subsequent recurrent CMV events [6], in agreement with another report studying SOT and HCT recipients [43]. We did not find a significant correlation found between ALC and risk for subsequent CMV event (Fig. 4). This discrepancy may be related to the higher rate of lymphocyte depletion (use of anti-lymphocyte induction therapy and HCT) in these reports, compared to our patient population.

Limitations of our study are its retrospective design, small sample size, host diversity and clinical scenarios in which this test was ordered. Our endpoint was initiation of antiviral for CMV guided by DNAemia or symptoms at clinician discretion, rather than CMV disease as defined by consensus criteria [44]. Nevertheless, clinicians frequently initiate treatment at high-level or rising CMV DNAemia before symptoms develop, therefore investigators have previously used this “real-world” outcome as a clinical endpoint when studying a CMV-CMI assay [26]. Last, providers were not blinded to test results, which might have influenced clinical thresholds to treat CMV DNAemia and choose observation over treatment with valganciclovir in patients with positive CMV-TCIP values. However, this does not seem to be the case, since patients with false positive CMV-TCIP results were classified as such because clinicians decided to start treatment. None of these patients had symptoms and signs clearly attributable to CMV infection, and at least two had evidence of controlling the infection (down-trending viremia), prior to initiation of valganciclovir. Also, peak CMV-VL was higher in cases with TCIP negative results, which argues against the clinicians having a lower threshold to treat CMV in such patients.

It should be noted that, besides CD69 and IFN-γ, other activation/memory markers like CCR7, CD45RO, CD27, CD62L and cytokines like TNF-α and IL2 of CD4+ and CD8+ T-cells may be helpful to further delineate CMV-specific TCI. In one small study of CMV R+ lung transplant recipients at risk for CMV infection, sensitivity was numerically lower but specificity higher for % TNF-α-producing CD8+ T-cells, while specificity was numerically higher for %TNF- α-producing CD4+ T-cells with the same sensitivity, compared to IFN-γ. IL2 had lower sensitivity and specificity compared to IFN-γ, whilst combining IFN-γ and IL2 did not improve predictive performance [45]. Future studies might help identify even more sensitive and specific CD4/8 “deep immunophenotyping” [46] and “polyfunctional signatures” [47], to predict protection against CMV.

## Conclusions

The CMV-TCIP assay, in particular %CMV-specific CD4+ T-cells, demonstrated good performance in predicting subsequent CMV events in immunocompromised patients at risk for CMV infection. Given the potential clinical utility of this assay, further validation in larger prospective studies is warranted.

## Data Availability

The datasets used and/or analyzed during the current study are available from the corresponding author on reasonable request.

## Abbreviations

ALC: Absolute lymphocyte count
AUC: Area Under the Curve
CI: Confidence intervals
CMI: Cell mediated immunity
CMV: Cytomegalovirus
FC: Flow cytometry
HCT: Hematopoietic cell transplant
HIV: Human Immunodeficiency Virus
ICS: Intracellular cytokine staining
IQR: Inter-quartile percentile range
NPV: Negative predictive values
OI: Opportunistic infections
PBMC: Peripheral blood mononuclear cells
PCR: Polymerase chain reaction
PPV: Positive predictive values
PTD: Post-transplant day
ROC: Receiver operating characteristic
SEB: *Staphylococcus aureus* Enterotoxin Type B
SOT: Solid organ transplantation
TCI/TCIP: T-cell immunity/ T-Cell Immunity Panel
UMMC: University of Maryland Medical Center
VL: Viral load
