# Correction to: Clinical experience with a novel assay measuring cytomegalovirus (CMV)-specific CD4+ and CD8+ T-cell immunity by flow cytometry and intracellular cytokine staining to predict clinically significant CMV events

**DOI:** 10.1186/s12879-020-4848-8

**Published:** 2020-02-11

**Authors:** Ralph Rogers, Kapil Saharia, Aditya Chandorkar, Zoe F. Weiss, Kendra Vieira, Sophia Koo, Dimitrios Farmakiotis

**Affiliations:** 1 Division of Infectious Diseases, Department of Internal Medicine, Warren Alpert Medical School of Brown University, 593 Eddy Street, Gerry House 111, Providence, RI 02903 USA; 2 Division of Infectious Diseases and Institute of Human Virology, University of Maryland School of Medicine, Baltimore, MD USA; 3 Division of Infectious Diseases, Department of Internal Medicine, Brigham and Women’s Hospital, Harvard Medical School, Boston, MA USA

**Correction to: BMC Infectious Diseases (2020) 20:58.**

**https://doi.org/10.1186/s12879-020-4787-4**

After publication of the original article [1], we were notified that an author’s family name has been erroneously spelled. Aditya Chandrokar should be replaced with Aditya Chandorkar.

The original article has been corrected as well.
